# Cancer Survivors Could Get Survival Benefits from Postdiagnosis Physical Activity: A Meta-Analysis

**DOI:** 10.1155/2019/1940903

**Published:** 2019-10-24

**Authors:** Yaohan Wang, Hongli Song, Yukun Yin, Li Feng

**Affiliations:** ^1^Traditional Chinese Medicine Department, National Cancer Center/Cancer Hospital, Chinese Academy of Medical Science and Peking Union Medical College, No. 17, Panjiayuannanli, Chaoyang District, Beijing 100021, China; ^2^China Academy of Chinese Medical Sciences, No. 16, Dongzhimen Inner South Street, Dongcheng District, Beijing 100700, China

## Abstract

**Background:**

Physical activity presents significant protection against death from cancer in the general population, so the global recommendations on physical activity for health are recommended by the WHO. While the recommendation is a guideline for general population, whether all cancer patients could get benefits from physical activity and whether the cancer patients who did not meet the requirement of the recommendation could get benefits from the physical activity, compared with the cancer patients with no physical activity, are unclear. Accordingly, we conducted a meta-analysis to identify whether the physical activity, even if low level of physical activity, could reduce the mortality of various cancer patients.

**Method:**

We conducted a systematic search of PubMed, Embase, and Cochrane Library for published cohorts and case-control studies of cancer survivors with physical activity comparing with no physical activity and reported outcomes of mortality through October 15, 2018. Two investigators independently reviewed the included studies and extracted relevant data. The effect estimate of interest was the hazard ratios (HRs).

**Results:**

There are 21811 participants in total in the nine studies, and 2386 cancer deaths in this meta-analysis. Among them, 1 was a case-control study and 8 were cohort studies. The meta-analysis results showed that physical activity was associated with a significantly reduced risk of mortality in cancer survivors, with a pooled HR and 95% CI of 0.66 (0.58∼0.73), reducing mortality by 34% and also suggested that low level of physical activity could reduce the mortality with an HR and 95% CI of 0.60 (0.50∼0.69).

**Conclusion:**

The results of this meta-analysis demonstrated that postdiagnosis physical activity, no matter the level of physical activity, could significantly reduce the mortality by 34%, compared with the no physical activity. At the same time, the results also suggested that cancer survivors undergoing low level of physical activity had a 40% reduction in mortality, which means that the cancer patients with poor ECOG need to do physical activity as much as they can, even if the amount of physical activity was low.

## 1. Introduction

Cancer is a major public health problem worldwide and the major leading cause of death in the world. Every year, 1,735,350 new cancer cases and 609,640 cancer deaths are projected to occur in the United States [1], and 4292,000 new cancer cases and 2814,000 cancer deaths would occur in China [2]. Over the past 25 years, the field of clinical oncology has experienced an exponential increase in research initiatives into the application of exercise for cancer patients or survivors [3]. And, many of the researches have proved that the highest level of physical activity presented significant protection against death from cancer in the general population [4]. So, the global recommendations on physical activity for health is recommended by the WHO, which recommends a minimum of 150 minutes of moderate-intensity physical activity or 75 minutes of vigorous-intensity physical activity per week or any equivalent combination for health benefits, and 300 minutes of moderate-intensity physical activity or 150 minutes of vigorous-intensity physical activity per week for additional health benefits [5]. While the recommendation is a guideline for the general population, whether patients with a noncommunicable chronic disease (NCD), especially with cancers, could benefit from this recommendation is unknown. So researches on NCD, especially on cancers, require further investigation. Recently, it has been demonstrated that physical activity significantly reduced the mortality in breast [6], colorectal [7], and prostate cancers [8]. And, it has been proved that compared with the low level of physical activities, the high level reduced cancer mortality more significantly [9]. However, some surveys have showed that not all cancer patients could adhere to physical activity guidelines [10, 11], and only 8% of cancer survivors could meet physical activity guidelines based on the objective accelerometry data; 11% of breast cancer survivors and 12% of endometrial cancer survivors could meet the guidelines [12]. Therefore, whether all cancer patients could benefit from physical activity and whether cancer patients who did not meet the requirements of the recommendation could benefit from the physical activity, compared with the cancer patients with no physical activity, are also unclear. Accordingly, we conducted a meta-analysis to identify whether the physical activity, even low level of physical activity, could reduce the mortality of various cancer patients.

## 2. Methods

### 2.1. Search Strategy

This meta-analysis was performed in accordance with the Preferred Reporting Items for Systematic Reviews and Meta-Analyses (PRISMA) guidelines [13]. Three databases, PubMed, Embase, and Cochrane Library, were searched from their inception to October 15, 2018, for cohort or case-control studies published in English that investigated the association between physical activity and mortality. The search terms included “exercise or exercises or sport or sports or physical activity or physical activities or yoga or qigong or taichi or exercise training or exercise trainings” and “cancer or cancers or neoplasm or neoplasms or tumor or tumors or carcinoma or carcinoma” and “mortality or mortality rate or death rate” (detailed search strategy is available in Supplementary Tables [Supplementary-material supplementary-material-1], [Supplementary-material supplementary-material-1], and [Supplementary-material supplementary-material-1]). The articles were searched by two authors independently, and if any disagreement, the third author would solve it.

### 2.2. Inclusion and Exclusion Criteria

1. Participants: adults aged 18 years and older with the diagnosis of cancer
2. Interventions: physical activity (e.g., leisure-time physical activity, recreational physical activity, exercise, sports, etc.) should be taken after the cancer diagnosis
3. Comparator: no physical activity
4. Outcome: mortality confirmed by follow-up or International Classification of Diseases (ICD) codes or records from government registration, presented as hazard ratios (HRs), risk difference (RD), risk ratio (RR), or odds ratio (OR), and associated 95% confidence intervals (CIs)
5. Study designs: cohort study or case-control studies

Studies were excluded if they (1) studied a population without cancer, (2) prediagnosis physical activity, (3) focused on cancer risk not cancer mortality, and (4) studies published not in English.

### 2.3. Data Extraction and Quality Assessment

Two investigators independently screened all the included studies to extract the following data: name of the first author, publication year, study design, country, study period, sample size, age at baseline, gender, duration of follow-up, adjustments/matching, intervention (amounts of physical activity at each level in different units), comparator, estimate of effect (reported as a HR, RD, RR, and OR) and the corresponding 95% CI for the association of physical activity with cancer mortality. The Newcastle–Ottawa Quality Assessment Scale (NOS) was employed to assess the quality of each of the included studies. Any discrepancy was resolved by discussion or by involving an arbiter.

### 2.4. Primary Outcomes

The mortality of cancer survivors after diagnosis.

### 2.5. Secondary Outcomes

The mortality of cancer survivors with a low level of physical activity after the diagnosis.

### 2.6. Statistical Analysis

The measure of interest was the HR (or the OR in case-control studies). Whenever available, we used multivariate-adjusted risk estimates. When possible, we chose no physical activity as the reference category. In a particular study, if more than one category fell in the exposure level considered, we combined the corresponding estimates using the method proposed by Hamling et al. [14]. This method was used to combine estimates using the same reference category or the same set of controls, taking into account correlation between the estimates. It used the adjusted estimates and the number of exposed and nonexposed subjects to derive a corresponding set of pseudonumber of cases and controls/subjects at risk consistent with the reported adjusted estimates. Assessment of heterogeneity was performed using Cochran's *Q* test and Higgins's *I*^2^; *I*^2^ >50% and a *P* value <0.10 suggested a significant heterogeneity [15, 16]. The random-effect model was used for the meta-analysis if there was a significant heterogeneity while the fixed effect model was used when the heterogeneity was not significant. Sensitivity analysis was performed by sequentially omitting each study to examine the robustness of the results. Potential publication bias was evaluated using Begg's funnel plot and Egger's test [17]. If significant publication bias existed, the trim-and-fill method was performed to validate the robustness of the meta-analysis results [18]. All statistical analysis was calculated via Stata 12.0. All two-tailed *P* values <0.05 were defined as statistical significance, except those for heterogeneity.

## 3. Results

### 3.1. Search Results

**Figure 1:**
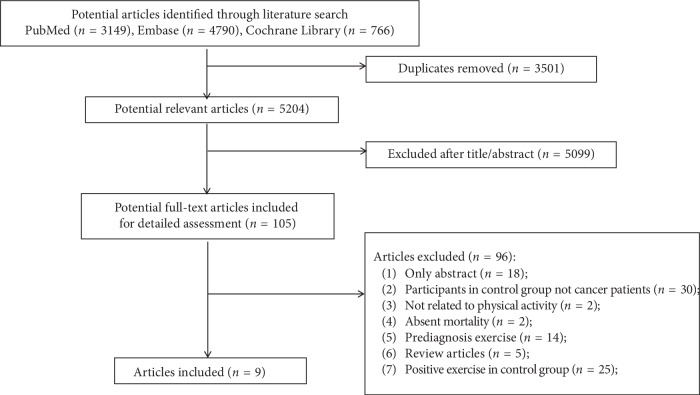
Flow diagram of study selection.

8705 articles met our search strategy from PubMed, Embase, and Cochrane Library. After the removal of duplicated articles, 5204 articles remained. And then reviewed the titles and abstracts, 5099 irrelevant articles were excluded. We conducted a full-text evaluation for the remaining 105 articles, and 96 articles were excluded. 96 articles were excluded for the following reasons: only abstract (*n* = 18), participants in the control group not cancer patients (*n* = 30), no related to physical activity (*n* = 2); absent mortality (*n* = 2); prediagnosis physical activity (*n* = 14); review articles (*n* = 5); and positive physical activity in the control group (*n* = 25). Finally, nine articles [19–27], involving a total of 21811 participants, were included in this meta-analysis (Figure 1).

### 3.2. Study Characteristics and Quality Assessment

There were 21811 participants in total in the nine studies and 2386 cancer deaths in this meta-analysis. Among them, 1 was a case-control study [20] and 8 were cohort studies [19, 21–27]. These studies were published between 2008 and 2018; one study was done in Japan [19], one in China [24], two in Australia [25, 26], and the other five all in America [20–23, 27]. And three studies provided data on the relationship between physical activity and mortality on breast cancer [21, 24, 27], the other six on esophageal and gastric cancer [19], ovarian cancer [20], colorectal cancer [22, 26], and various cancer [23, 25], respectively. In the meta-analysis, eight studies adopted recreational physical activity or regular exercise as the intervention [19–22, 24–27], such as dancing, biking, or jogging, and one study adopted resistance exercise [23]. All studies were matched or adjusted, eight of them adjusted for at least age and sex [19–25, 27], and one study did not mention the details [26]. And all studies reported mortality presented as hazard ratios (HRs). The characteristics of the interventions of included articles are listed in Table 1. The overall quality score ranged from 7 to 9 based on the Newcastle–Ottawa scale in all nine studies (Table 2).

### 3.3. Primary Outcomes

**Figure 2:**
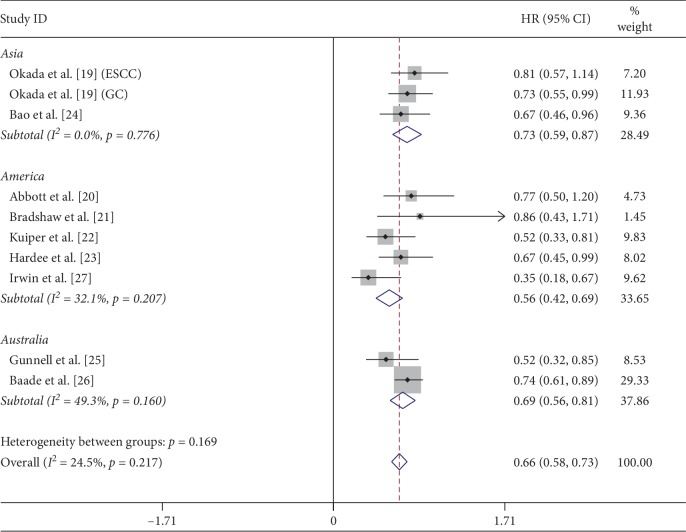
Meta-analysis of mortality.

Nine studies with 21811 participants were included in the meta-analysis of mortality, with 2386 death cases. Among them, 12363 participants did no physical activity, 9179 participants did low or high levels of physical activity, and 1386 participants had no information about physical activity. In the nine studies, only 2 studies offered HRs and 95% CIs compared with the reference group (no physical activity group), 7 studies offered HRs and 95% CIs of different levels of physical activity, not the total HRs and 95% CIs, compared with the reference group. The meta-analysis results showed that the total physical activity, no matter high or low level, was associated with a significantly reduced risk of mortality in cancer survivors, with a pooled HR and 95% CI of 0.66 (0.58∼0.73, *P* ≤ 0.001) (Figure 2). The physical activity reduced mortality by 34% in cancer survivors. And the heterogeneity was not significant (*P*=0.217, *I*^2^ = 24.5%). In the subgroup analysis, physical activity in America decreases the mortality with 44% reduction (HR = 0.56, 95% CI = 0.42∼0.69, *P* ≤ 0.001), while Australia with 31% reduction (HR = 0.69, 95% CI = 0.56∼0.81, *P* ≤ 0.001) and Asia with 27% reduction (HR = 0.73, 95% CI = 0.59∼0.88, *P* ≤ 0.001).

Sensitivity analysis by sequentially omitting each study was employed. We found that the study by Irwin et al. [27] influenced the pooled HR. Removing this study yielded an HR and 95% CI of 0. 69 (0.61∼0.77), with a low heterogeneity (*P*=0.729, *I*^2^ = 0.0%). And there was no publication bias according to the funnel plot using both Begg's test (*P*=0.152) and Egger's test (*P*=0.345).

### 3.4. Secondary Outcomes

**Figure 3:**
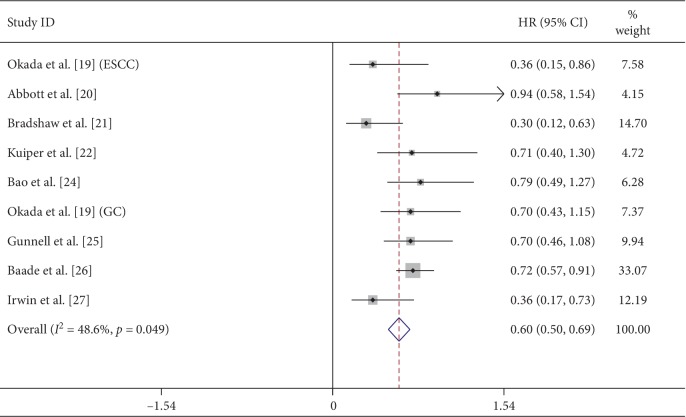
Meta-analysis of low physical activity mortality.

Among the nine studies, eight of them offered the mortality of the low-level physical activity with 18958 participants and 2265 death cases. Calculation using the fixed effects model yielded a pooled HR and 95% CI of 0.60 (0.50∼0.69, *P* ≤ 0.001) (Figure 3), with a significant heterogeneity (*P*=0.049; *I*^2^ = 48.6%). To explore the source of heterogeneity, we performed an analysis in the subgroup by region and found that the America group had a significant heterogeneity (*P*=0.072; *I*^2^ = 57.2%). So sensitivity analysis by sequentially omitting each study was employed. We found that the study by Irwin et al. [27] substantially influenced the pooled HR. Removing this study yielded an HR and 95% CI of 0. 70 (0.59∼0.81), with a low heterogeneity (*P*=0.661, *I*^2^ = 0.0%). The cause for this performance may be that the participants included 366 black women among 1183 women.

## 4. Discussion

In this meta-analysis, we found that physical activity after the cancer diagnosis, no matter the total physical activity or the low physical activity, could significantly reduce the mortality by 34% and 40%, respectively, compared the no physical activity.

First, it was known that physical activity as one of the important lifestyle factors could reduce the mortality of coronary heart disease [28], chronic obstructive pulmonary disease (COPD) [29], and diabetes [30], and researches had shown that physical activity also could decrease the cancer incidence and mortality in the general population [4]. For colon and breast cancer, it has been demonstrated that postdiagnosis physical activity could protect from death, but whether all cancer survivors could benefit from postdiagnosis physical activity was still unclear. This meta-analysis included various cancer survivors, and the result revealed that no matter what kind of physical activity both could make cancer survivors benefit in reducing mortality. Because of the poor quality of life of some cancer survivors, the recommendation of WHO was not available, and whether these patients should do physical activity puzzled us. Therefore, for these cancer survivors, the meta-analysis also tried to answer the question: these patients with poor quality of life could get benefits from the low-level physical activity. In other words, doing physical activity is better than not doing.

Second, in the subgroup analysis, we found that cancer survivors in America benefited more from postdiagnosis physical activity than cancer survivors in Asia by 18%. It may be related to the following points: (1) The researches conducted in Asia were few in this meta-analysis. In the nine studies, only 2 studies with 11742 participants were conducted in Asia, while 5 studies with 6577 in America. Even though the participants in Asia were more than those in America, the data collected from the 2 studies including esophageal, gastric, and breast cancers from 2002 to 2007 were less universality compared with the data from America which were collected from 5 studies and included various cancers from 1987 to 2015. So the data from Asia might be the cause leading to 18% difference. (2) Different regions: in Asia, the people almost are Asian, and Caucasian is the main race in America. Different races have different cultures and habits. The result of meta-analysis might suggest that the same recommendation could not be applied all cancer survivors in the word. We need different physical activity recommendations for different people to make them benefit more from it in future. In addition, the Australia group had no signification with Asia group; the cause may be that the two studies in Australia had a high heterogeneity (*P*=0.16, *I*^2^ = 49.3%) which influenced the result in the end.

Third, the secondary outcomes showed that low-level physical activity could reduce mortality by 40%. The result suggested that low-level physical activity could also protect cancer patients from death, which encouraged the cancer patients who had poor ECOG do physical activity as much as they can, even if the amount of physical activity was low.

The mechanism about physical activity reducing the mortality in cancer survivors was unknown, but some researches showed that it could improve survival by regulating the immune function, inflammation, modulating the insulin pathway, and epigenetic changes. Physical activity had direct effects on the cellular immune system. Cytotoxic immune cells were mobilized to the circulation through the stress-induced shear stress and adrenergic signaling during exercise performance [31]. Once mobilized, these cytotoxic immune cells survey the body for transformed cells as immunological targets. It was shown that exercise-mediated inhibition of tumor growth by more than 50% reduction in mouse models was driven by an epinephrine-dependent mobilization of NK cells to the circulation and increased intratumoral immune cell infiltration [32]. Moreover, physical activity leads to epigenetic changes that could have beneficial effects in cancer patients. This epigenetic alteration in cancer cells would cause the cell to grow and break down, resulting in a tumor. Physical activity to reduce this mutation and even reverse epigenetics had been shown to increase the level of expression of the tumor suppressor gene, reduce the level of oncogenesis [33, 34], and reduce cancer cells with abnormal DNA methylation patterns, including hypermethylation in tumor-suppressor gene-promoter regions and hypomethylation in promoter regions of oncogenes [33]. The effect of exercise on DNA-methylation patterns led to an increase in the expression of the gene associated with tumor suppression and decreases the expression of oncogenes [33, 34]. Hypermethylation in tumor promoter regions was a suppressor of APC and RASSF1A genes. Exercise had been shown to be a reducing agent, and even a suppressant of hypermethylation, as well as in reducing and even reversing the hypermethylation of APC and RASSF1A promoters, reducing their risk of cancer [35].

There are some limitations to this meta-analysis. First, even though the meta-analysis had shown that postdiagnosis physical activity, no matter the level of it, could significantly reduce the cancer survivors' mortality, we did not compare the high level of physical activity with the low or moderate level. Recently, some researches suggested that high level of physical activity reduced the mortality more significantly than low or moderate level, but whether it had statistical differences between them was unclear. So, we should make a meta-analysis between high and moderate or low levels of physical activity in cancer survivors in future. Second, in the subgroup meta-analysis, we assessed the association between physical activity and cancer mortality differed by region, but we did not assess the association between physical activity and cancer mortality differed by gender, age, or race because of lack of variation among the studies. Third, the studies we searched from PubMed, Embase, and Cochrane Library were published in English. We did not include studies found in other databases, not written in English, or published as a conference abstract. We acknowledge this as a limitation.

## 5. Conclusion

The results of this meta-analysis demonstrated that postdiagnosis physical activity, no matter the level of the physical activity, significantly reduced the mortality by 34%, compared with no physical activity. At the same time, the results also suggested that cancer survivors undergoing a low level of physical activity had a 40% reduction in mortality, which means that the cancer patients with poor ECOG need to do physical activity as much as they can, even if the amount of physical activity is low.

## Figures and Tables

**Table 1:** Characteristics of the studies included in the meta-analysis.

	Study	Year	Design	Country	Study period	Cancer types	Age	Gender	Study size	Follow-up	Adjustments or match	Intervention	Comparator
1	Okada et al. [19]	2017	Cohort	Japan	2003–2007	Esophageal and gastric cancer	24–95	Male Female	1604 (1053/102/338/111); death: 213 9620 (6392/516/2105/607); death: 603	ESCC: 4.4 years GC: 6.1 years	Sex, age, year of diagnosis, BMI, smoking history, alcohol drinking history, and stage	1-2 times/week ≥3 times/week Unknown	No habit

2	Abbott et al. [20]	2018	Case-control	USA	2010–2015	Ovarian cancer	20–79	Female	264 (130/90/44); death: 80	42.7 months	Age, stage, geographic region, number of comorbid conditions, education, and income. RPA after diagnosis is additionally adjusted for prediagnosis RPA (0, >0–9, >9 MET-hours/week)	>0–9 MET-hours/week >9 MET-hours/week	0

3	Bradshaw et al. [21]	2014	Cohort	USA	1996-1997	Breast cancer	25–91	Female	1423 (349/30/181/668); death: 420	5 years	Missing data: PA, chemotherapy, and tumor size, which assumes that the missing data mechanism for PA is ignorable	0.1–9.0 MET h/week >9 MET h/week Missing	0

4	Kuiper et al. [22]	2012	Cohort	USA	1993–1998	Colorectal cancer	50–79	Female	1339 (234/166/350/312/277); death: 171	11.9 years	Adjusted for age at diagnosis, study arm, BMI, tumor stage, ethnicity, education, alcohol, smoking, and hormone therapy use	>0–2.9 MET-hours/week 3.0–8.9 MET-hours/week 9.0–17.9 MET-hours/week ≥18 MET-hours/week	0

5	Hardee et al. [23]	2014	Cohort	USA	1987–2002	Cancers	18–81 (54.4)	Male Female	2863 (PA: 1117/1746RE: 1612/1251); death: 121	7.3 years	Age, gender, and examination year, body mass index, current smoking (yes or no), heavy drinking (yes or no), hypertension (present or not), diabetes (present or not), hypercholesterolemia (yes or no), and parental history of cancer (yes or no)	RE: yes	RE: no
6	Bao et al. [24]	2015	Cohort	China	2002–2006	Breast cancer	20–75	Female	518 (175/343); death: 128	9.1 years	Age at diagnosis (continuous variable), education (<middle school, middle school, high school, >high school), marital status, Charlson comorbidity index (0, ≥1), menopausal status (yes, no), BMI at baseline (<18, 18–24.99, 25–29.99, ≥30), soy protein intake (Q1–Q4), tea consumption at baseline (yes, no), chemotherapy (yes, no), radiotherapy (yes, no), and TNM stage (I, II, III, unknown)	Yes	No

7	Gunnell et al. [25]	2017	Cohort	Australia	2004–2011	Cancers	68	Male Female	1667 (439/460/384/384); death: 135	8.8 years	Age at survey, sex, smoking category, long-term risky drinking category, body mass index category, daily fruit and vegetable intake, survey year, self-reported diabetes, SF-8 mental health component score, SF-8 physical health component score, and previous cancer type	<150 min LTPA/week 150–359 min LTPA/week 360 + min LTPA/week	No LTPA

8	Baade et al. [26]	2011	Cohort	Australia	2003–2008	Colorectal cancer	20–70+	Male Female	1825 (748/484/593); death: 462	4.9 years	Not mentioned	Insufficiently active Sufficiently active	Sedentary

9	Irwin et al. [27]	2008	Cohort	USA	1995–2004	Breast cancer	>18	Female	688 (114/297/277); death: 53	2.5 years	Age, race, disease stage, initial treatment, tamoxifen use, body mass index, and fruit/vegetable servings per day	>0–8.9 MET-h/wk ≥9 MET-h/wk	0 MET-h/wk

ESCC: esophageal cancer; GC: gastric cancer; PA: physical activity; RE: resistance exercise.

**Table 2:** Study quality assessment (Newcastle–Ottawa scale).

Study	Selection				Comparability	Outcome			Total no. of stars
	Exposed cohort	Nonexposed cohort	Ascertainment of exposure	Outcome of interest		Assessment of outcome	Length of follow-up	Adequacy of follow-up	
*Cohort studies*									
Okada et al. [19]	^*∗*^	^*∗*^	^*∗*^	^*∗*^	^*∗∗*^	^*∗*^	^*∗*^	^*∗*^	9
Bradshaw et al. [21]	^*∗*^	^*∗*^	^*∗*^	^*∗*^	^*∗∗*^	^*∗*^	^*∗*^	^*∗*^	9
Kuiper et al. [22]	^*∗*^	^*∗*^	^*∗*^	^*∗*^	^*∗∗*^	^*∗*^	^*∗*^	—	8
Hardee et al. [23]	^*∗*^	^*∗*^	—	^*∗*^	^*∗∗*^	^*∗*^	^*∗*^	^*∗*^	8
Bao et al. [24]	^*∗*^	^*∗*^	^*∗*^	^*∗*^	^*∗∗*^	—	^*∗*^	^*∗*^	8
Gunnell et al. [25]	^*∗*^	^*∗*^	—	^*∗*^	^*∗∗*^	^*∗*^	^*∗*^	^*∗*^	8
Baade et al. [26]	^*∗*^	^*∗*^	^*∗*^	^*∗*^	—	^*∗*^	^*∗*^	^*∗*^	7
Irwin et al. [27]	—	^*∗*^	^*∗*^	^*∗*^	^*∗∗*^	^*∗*^	^*∗*^	^*∗*^	8

*Case-control studies*									
Study	Selection				Comparability	Exposure			Total no. of stars
	Definition of cases	Representativeness of cases	Selection of controls	Definition of controls		Assessment of outcome	Method of ascertainment	Nonresponse rate	

Abbott et al. [20]	^*∗*^	^*∗*^	^*∗*^	^*∗*^	^*∗∗*^	^*∗*^	^*∗*^	—	8

## Supplementary Materials

Supplementary Materials Table S1: search strategy used in PubMed. Table S2: search strategy used in Embase. Table S3: search strategy used in Cochrane Library.
