# Supplementary material for: Cancer Survivors Could Get Survival Benefits from Postdiagnosis Physical Activity: A Meta-Analysis
Source: Evid Based Complement Alternat Med. 2019 Oct 24;2019:1940903. doi: 10.1155/2019/1940903 (PMC6854247; doi:10.1155/2019/1940903)
Supplement: Supplementary Materials — Table S1: search strategy used in PubMed. Table S2: search strategy used in Embase. Table S3: search strategy used in Cochrane Library. [file 1940903.f1.doc]

Table S1: Search Strategy Used in PubMed

| 1 | ((((((((((exercise[Title/Abstract]) OR exercises[Title/Abstract]) OR Sport[Title/Abstract]) OR Sports[Title/Abstract]) OR physical activity[Title/Abstract]) OR physical activities[Title/Abstract]) OR yoga[Title/Abstract]) OR qigong[Title/Abstract]) OR taichi[Title/Abstract])) OR (("Exercise"[Mesh]) OR "Sports"[Mesh])OR ((exercise training[Title/Abstract]) OR (exercise trainings[Title/Abstract])) | [495419](https://www.ncbi.nlm.nih.gov/pubmed/?cmd=HistorySearch&querykey=3) |
| --- | --- | --- |
| 2 | (((((((((cancer[Title/Abstract]) OR cancers[Title/Abstract]) OR Neoplasm[Title/Abstract]) OR Neoplasms[Title/Abstract]) OR tumor[Title/Abstract]) OR tumors[Title/Abstract]) OR carcinoma[Title/Abstract]) OR carcinoma[Title/Abstract])) OR "Neoplasms"[Mesh] | [3804528](https://www.ncbi.nlm.nih.gov/pubmed/?cmd=HistorySearch&querykey=54) |
| 3 | ("Mortality"[Mesh]) OR (((mortality) OR mortality rate) OR death rate) | [1234764](https://www.ncbi.nlm.nih.gov/pubmed/?cmd=HistorySearch&querykey=1) |
| 4 | 1 AND 2 AND 3 | 3149 |

Table S2: Search Strategy Used in Embase

| 1 | 'exercise'/exp OR 'sport'/exp OR 'physical activity'/exp OR 'exercise':ab,ti OR 'exercises':ab,ti OR 'sport':ab,ti OR 'sports':ab,ti OR 'physical activity':ab,ti OR 'physical activities':ab,ti OR 'exercise training':ab,ti OR 'exercise trainings':ab,ti | [894592](https://www.ncbi.nlm.nih.gov/pubmed/?cmd=HistorySearch&querykey=3) |
| --- | --- | --- |
| 2 | 'malignant neoplasm'/exp OR 'cancer':ab,ti OR 'cancers':ab,ti OR 'neoplasm':ab,ti OR 'neoplasms':ab,ti OR 'tumor':ab,ti OR 'tumors':ab,ti OR 'carcinoma':ab,ti OR 'carcinomas':ab,ti | [4741380](https://www.ncbi.nlm.nih.gov/pubmed/?cmd=HistorySearch&querykey=54) |
| 3 | ("Mortality"[Mesh]) OR (((mortality) OR mortality rate) OR death rate) | [1176222](https://www.ncbi.nlm.nih.gov/pubmed/?cmd=HistorySearch&querykey=1) |
| 4 | 1 AND 2 AND 3 | 4790 |

Table S3: Search Strategy Used in Cochrane Library

| 1 | [exercise] explode all trees OR [sports] explode all trees OR (exercises) ti,ab,kw OR (physical activities) ti,ab,kw OR (sports) ti,ab,kw OR (taiqi) ti,ab,kw OR (exercise) ti,ab,kw OR (physical activity) ti,ab,kw OR (sport) ti,ab,kw OR (yoga) ti,ab,kw OR (qigong) ti,ab,kw | 120042 |
| --- | --- | --- |
| 2 | [neoplasms] explode all trees OR (cancer) ti,ab,kw OR (cancers) ti,ab,kw OR ('neoplasm) ti,ab,kw OR (neoplasms) ti,ab,kw OR (tumor) ti,ab,kw OR (tumors) ti,ab,kw OR (carcinoma) ti,ab,kw OR (carcinomas) ti,ab,kw | 200988 |
| 3 | [mortality] explode all trees OR (mortality rate) ti,ab,kw OR (mortality) ti,ab,kw OR (death rate) ti,ab,kw | 10426 |
| 4 | 1 AND 2 AND 3 | 766 |
